# Association between number of children and incident heart disease and stroke in parents – results from the Survey of Health, Ageing and Retirement in Europe (SHARE)

**DOI:** 10.1186/s12889-023-17254-7

**Published:** 2023-11-24

**Authors:** Carolin Girschik, Susanne Stolpe, Bernd Kowall

**Affiliations:** Institute of Medical Informatics, Biometry and Epidemiology (IMIBE), University Hospital of Essen, Hufelandstraße 55, 45147 Essen, Germany

**Keywords:** Parity, Number of children, Heart disease, Stroke, Cardiovascular disease

## Abstract

**Background:**

In former studies, parity was associated with adverse cardiovascular outcomes in parents. This study aims to extend the limited existing data regarding the association between the number of children and heart disease and/or stroke in a large longitudinal study in different European countries in both men and women.

**Methods:**

For 42 075 subjects (18 080 men, 23 995 women; median age 58 years (interquartile range: 53 to 65)) from 19 European countries and Israel in the Survey of Health, Ageing and Retirement in Europe (SHARE), odds ratios (OR) for the association between number of children and incident self-reported heart disease and/or stroke (HDS) were estimated using logistic regression analyses. Persons with one or two children were used as reference. The final model was adjusted for baseline age, sex, education, region, and marital status. All analyses were stratified by sex.

**Results:**

Women with seven or more children had the highest OR for the association between the number of children and incident HDS (OR = 2.12 [95% CI: 1.51 to 2.98]), while men with six children showed the highest OR (OR = 1.62 [1.13 to 2.33]). Stratified by education, across all education levels, men and women with five or more children had the highest ORs for this association. The highest OR was observed in both women and men in the group with primary education (OR = 1.66 [1.29 to 2.15] and OR = 1.60 [1.19 to 2.14], respectively). Stratified by region, both men and women with five or more children showed the highest ORs in Southern Europe (OR = 2.07 [1.52 to 2.82] and OR = 1.75 [1.25 to 2.44], respectively).

**Conclusion:**

In this long-term follow-up study in various countries in Europe and Israel we found a positive association between number of children and incident HDS. This association was more pronounced in lower educated subjects and showed regional variations.

**Supplementary Information:**

The online version contains supplementary material available at 10.1186/s12889-023-17254-7.

## Background

Parity can affect parental health in a variety of ways. While biological changes due to pregnancy, childbirth, and breastfeeding are associated with cardiovascular risks in women, social mechanisms that affect both sexes have also been suggested [1–4]. For this reason, the health of both women and men is affected, albeit by different underlying factors. Moreover, a different workload with child rearing between the sexes cannot be ruled out. Rather than childbirth, the number of children appears to have an impact on cardiovascular risk. While some studies describe a J-shaped relationship [5–8], other studies either report the lowest risk of heart disease in childless individuals and an increased risk only in higher parity categories [9–13], or no association [14–16]. Similar diverse results were observed for the association between the number of children and stroke [12, 13, 15]. On closer inspection, the studies to date are very heterogeneous. There are studies that looked at women only [6, 11–15], and studies with a small number of participants [11, 12, 16]. Furthermore, a majority of the studies were conducted in the United States [9, 11, 14–16], the United Kingdom [7, 10], China [5, 13], and Scandinavian countries [6, 12]. Regional comparisons are currently lacking and could provide important indications of different structural conditions between countries. In addition, a lower educational level is associated with both a higher number of children and adverse economic and social characteristics that may lead to a different cardiovascular risk between individuals [17, 18].

Our aim is to extend the literature on the association between the number of children and incident heart disease and/or stroke (HDS) in both men and women in a population from various countries in Europe and Israel. To explore whether educational attainment or region is independently associated with HDS, sex-stratified subgroup analyses of this association will be conducted.

## Methods

### Sample

We used data from the Survey of Health, Ageing and Retirement in Europe (SHARE) [19]. This longitudinal, harmonized panel contains data on health, socio-economic situation, and social networks of elderly people in 28 European countries and Israel. Our analyses were based on data from waves 1 to 7 of this survey, which were collected between 2004 and 2017. We used participants’ answers from the interview on children in each wave to define the exposure. The outcome was defined using information from the regular SHARE interviews on physical health and—in case of death of a participant – the standard SHARE end-of-life interview with the participant’s relative in waves 5 to 7.

The working sample consisted of 133 198 participants with at least one valid interview in waves 1 to 7 (except for wave 3). Participants without information on the number of children were excluded from further analyses (*n* = 18 677). After that, all participants were excluded who reported at study entry having had HDS (*n* = 17 543), taking heart medication (drugs for high blood cholesterol, high blood pressure, coronary or cerebrovascular diseases, and other heart diseases) (*n* = 38 563), or who had given an invalid answer regarding this (*n* = 441) when first answering the question. After further exclusion of subjects with no follow-up data about HDS (*n* = 15 899), the study population consisted of 42 075 subjects (18 080 men; 23 995 women) aged 24 to 102 years (Supplementary Fig. 1). Of these, only individuals without missing values on marital status and with an educational level according to the 1997 International Standard Classification of Education (ISCED 97), codes 0 to 6, were considered in the main analysis (*n* = 41 699).

### Heart disease and stroke

The outcome ‘heart disease and/or stroke (HDS)’ was self-reported and assessed via questionnaires. All participants were shown a list of 16 health outcomes and asked at wave 1: ‘Has a doctor ever told you that you had any of the conditions on this card?’. In waves 2 to 7 (except wave 3), the question was changed as follows:’Has a doctor ever told you that you had/Do you currently have any of the conditions on this card?’ The outcome HDS was defined as ‘heart attack including myocardial infarction or coronary thrombosis or any other heart problem including congestive heart failure’ and/or ‘stroke or cerebral vascular disease’. In addition, in the case of the death of a participant, in waves 5, 6, and 7, end-of-life interviews were conducted with family or household members who were asked: What was the main cause of [his/her] death? Variables were coded dichotomously, meaning with or without disease.

### Number of children

The exposure ‘number of children’ was self-reported by one family member. The family respondent was asked the following question: ‘How many children do you have that are still alive? Please count all natural children, fostered, adopted and stepchildren.’ The first report of the number of children was taken as the exposure variable and adopted for the partner. Partners of the family respondent were identified using a created CoupleID. Depending on the analysis strategy, the self-reported number of children was used continuously, categorized as none, 1, 2, 3, 4, 5, 6, and 7 + children, or categorized in groups of 1–2, 3–4, and 5 + children. The 2-child and 1–2-child groups were used as references because the incidence of HDS was lowest in this group.

### Covariates

The selection of covariates for statistical adjustment was based on previous literature [20–24]. All covariates were self-reported by the respondents in baseline and follow-up interviews. Covariates included baseline age, sex, education, region, marital status, age at first birth, and health status until age 15. For descriptive analyses, respondents were categorized into one of seven age categories (< 35, 35–44, 45–54, 55–64, 65–74, 75–84, ≥ 85) based on their reported age. Since both exposure and outcome were expected to be linearly related to age, baseline age was continuously included in the model. All analyses were sex-stratified.

The level of education reported by respondents in the baseline interview was categorized according to ISCED 97 and included in the main model (code 0–6). For further analyses, education was grouped into: ‘primary’ (pre-primary and primary education), ‘secondary’ (lower secondary, upper secondary, and post-secondary non-tertiary education), and ‘tertiary’ (first and second stages of tertiary education). Subjects with ‘no degree or other’ were excluded from statistical analyses (*n* = 190).

The final study population consisted of participants from 20 countries. In the model, these countries were categorized into four regions: ‘Northern Europe’ (Sweden, Denmark), ‘Southern Europe’ (Spain, Italy, Greece, Israel, Portugal), ‘Western Europe’ (Austria, Germany, Netherlands, France, Switzerland, Belgium, Luxembourg), and ‘Eastern Europe’ (Czech Republic, Poland, Hungary, Slovenia, Estonia, Croatia).

The first report of marital status was categorized into three groups for analysis: ‘married’ (married and living together or separated from spouse or registered partnership), ‘never married’, and ‘divorced or widowed’.

The variable ‘age at first birth’ was calculated from the first valid information of the year of birth of the first child, and the year of birth of the participant given in the baseline interview. If the age at first birth was less than 12 years (*n* = 119) or if a person reported no children but still had a child's birth year noted (*n* = 296), it was set as missing. For descriptive analyses, the variable ‘age at first birth’ was categorized into < 20, 20–29, 30–39, 40–49, and ≥ 50 years and continuously included in the model.

### Statistical analyses

Baseline characteristics were reported for the whole study population and separately for men and women. Sex-specific risks of incident cases of HDS were reported by number of children, by categories of baseline age and age at first birth, by health status until age 15, by education, marital status, and region. The frequency of reporting different numbers of children during the study period was calculated.

To examine the association between number of children and parental incident HDS logistic regression analyses were performed and odds ratios (OR) with 95%-confidence intervals (95% CI) estimated.

To control for potential confounding, two models were conducted in addition to the unadjusted crude model: Model 1 adjusted for baseline age and sex; and Model 2 (main model) additionally adjusted for education, region, and marital status. There were 11 943 and 8 370 missing data for age at first birth and health status until age 15, respectively, but these variables were potential confounders. However, adding these variables to Model 2 led to a change estimate of < 5%, and therefore were not included in the adjustment set. All models were additionally stratified by sex. Having two children was used as reference. Further sensitivity analyses were carried out. Model 2 was also calculated with people included in the analyses who were only taking medication for high blood pressure or elevated cholesterol to check that no large group of people was excluded from the analyses that would strongly influence the outcome. Furthermore, a sensitivity analysis was performed for model 2 in which the influence of the number of children on the outcomes heart disease and stroke was calculated separately.

Stratified analyses by sex and education or region were conducted to estimate ORs and 95% CIs for the association between number of children (0, 1–2, 3–4 or 5 +) and parental incident HDS. All of these analyses were adjusted for baseline age, marital status, and, depending on the model, for region or education. Persons with one or two children were used as reference.

All statistical analyses were performed using SAS version 9.4 (SAS Institute Inc., Cary, NC, USA).

## Results

Of the 42 075 SHARE participants, 43% were men (Table 1). The most frequent category of children were two children (42.1% men and 41.8% women). Slightly more men than women reported having no children (11.0% vs. 9.1%). At the birth of the first child, men were two years older than women (median age: 27, Q1: 25; Q3: 31 vs. 25, Q1: 22; Q3: 28). Furthermore, men and women had similar levels of education, although slightly more women had completed primary and lower secondary education (16.7% and 17.1% vs. 14.8% and 15.9%), while men were slightly more likely to have completed upper secondary education (35.8% vs. 33.5%). More men were married or living in a registered partnership than women (81.0% vs. 71.7%), while more women reported being divorced or widowed compared to men (22.4% vs. 11.6%). In addition, men and women were evenly distributed among the regions, and health status until age 15 was comparable between both sexes. Due to changing relationships over the study period, some participants reported different numbers of children when comparing the waves. Variation in the number of children reported during the study period was present in 11.7% of subjects (Supplementary Table 1).

**Table 1:** Characteristics of the study population at baseline

	**All (*n* = 42 075)**	**Men (*n* = 18 080) 43.0%**	**Women (*n* = 23 995) 57.0%**
**Baseline age (in years)**	58 [53; 65]	59 [54; 66]	57 [52; 65]
**BMI**^**a**^	25.8 ± 4.1 25.3 [23.1; 28.0]	26.3 ± 3.6 25.9 [24.0; 28.3]	25.4 ± 4.5 24.8 [22.4; 27.7]
**Number of children**	2.1 ± 1.4	2.1 ± 1.4	2.2 ± 1.4
0	4 175 (9.9)	1 996 (11.0)	2 179 (9.1)
1	7 239 (17.2)	2 944 (16.3)	4 295 (17.9)
2	17 633 (41.9)	7 603 (42.1)	10 030 (41.8)
3	8 141 (19.4)	3 427 (19.0)	4 714 (19.7)
4	2 883 (6.9)	1 232 (6.8)	1 651 (6.7)
5	1 073 (2.6)	481 (2,7)	592 (2.5)
6	471 (1.1)	194 (1.1)	277 (1.2)
≥ 7	460 (1.1)	203 (1.1)	257 (1.1)
**Age at first birth (in years)**^**b**^	26.7 ± 5.5 26 [23; 30]	28.4 ± 5.6 27 [25; 31]	25.4 ± 5.1 25 [22; 28]
**Education (ISCED 97)**^**c**^			
0—Pre-primary education	1 520 (3.6)	630 (3.5)	890 (3.7)
1—Primary education	6 637 (15.8)	2 661 (14.8)	3 976 (16.7)
2—Lower secondary education	6 943 (16.6)	2 861 (15.9)	4 082 (17.1)
3—Upper secondary education	14 439 (34.5)	6 441 (35.8)	7 998 (33.5)
4—Post-secondary non-tertiary education	1 922 (4.6)	829 (4.6)	1 093 (4.6)
5—First stage of tertiary education	9 876 (23.6)	4 308 (23.9)	5 568 (23.3)
6—Second stage of tertiary education	376 (0.9)	209 (1.2)	167 (0.7)
No degree/ other	190 (0.5)	77 (0.4)	113 (0.5)
**Marital status**^**d**^			
Married	31 785 (75.7)	14 618 (81.0)	17 167 (71.7)
Never married	2 752 (6.6)	1 343 (7.4)	1 409 (5.9)
Divorced / widowed	7 459 (17,8)	2 089 (11.6)	5 370 (22.4)
**Region (as defined in methods)**			
Northern Europe	5 533 (13.2)	2 424 (13.4)	3 109 (13.0)
Southern Europe	10 400 (24.7)	4 447 (24.6)	5 953 (24.8)
Western Europe	16 896 (40.2)	7 203 (39.8)	9 693 (40.4)
Eastern Europe	9 246 (22.0)	4 006 (22.2)	5 240 (21.8)
**Health status until age 15**^**e**^			
Excellent	11 702 (34.7)	5 215 (36.3)	6 487 (33.6)
Very good	10 740 (31.9)	4 529 (31.5)	6 211 (32.1)
Good	8 090 (24.0)	3 361 (23.4)	4 729 (24.5)
Fair	2 175 (6.5)	879 (6.1)	1 296 (6.7)
Poor	755 (2.2)	279 (1.9)	476 (2.5)
Health varied a great deal	106 (0.3)	37 (0.3)	69 (0.4)
Don’t know	137 (0.4)	80 (0.6)	57 (0.3)

Median [first quartile; third quartile]; mean ± standard deviation; n (%)

^a^1 013 Missings (Men: 320; Women: 693)

^b^11 943 Missings (Men: 5 500; Women: 6 443)

^c^172 Missings (Men: 64; Women: 108)

^d^79 Missings (Men: 30; Women: 49)

^e^8 370 Missings (Men: 3 700; Women: 4 670)

Risks of incident cases of HDS were highest in men and women with seven or more children (21.7% and 19.5%), and lowest in men without children (12.2%) and women with two children (7.3%) (Table 2). With increasing educational level, the risk of incident HDS decreased in both sexes (pre-primary education: 16.6% vs. second stage of tertiary education: 6.9%). Subjects from Western Europe showed the lowest risk for incident HDS (9.3%) compared to Northern (10.7%), Southern (11.5%), and Eastern Europe (11.4%).

**Table 2:** Incidence of HDS by characteristics of the study population at baseline stratified by sex

	**All (*n* = 42 075)**	**Men (*n* = 18 080)**	**Women (*n* = 23 995)**
	Heart disease and/or stroke 4 408 (10.5)	Heart disease and/or stroke 2 375 (13.1)	Heart disease and/or stroke 2 033 (8.5)
	N (%)	N (%)	N (%)
**Number of children**			
0	459 (11.0)	243 (12.2)	216 (9.9)
1	756 (10.4)	377 (12.8)	379 (8.8)
2	1 676 (9.5)	943 (12.4)	733 (7.3)
3	859 (10.6)	466 (13.6)	393 (8.3)
4	331 (11.5)	176 (14.3)	155 (9.4)
5	153 (14.3)	84 (17.5)	69 (11.7)
6	80 (17.0)	42 (21.7)	38 (13.7)
≥ 7	94 (20.4)	44 (21.7)	50 (19.5)
**Baseline age**			
< 35 years	0 (0.0)	0 (0.0)	0 (0.0)
35–44 years	20 (3.6)	4 (6.6)	16 (3.2)
45–54 years	712 (5.2)	418 (7.7)	294 (3.5)
55–64 years	1 470 (9.1)	827 (11.4)	643 (7.3)
65–74 years	1 340 (16.7)	739 (19.3)	601 (14.3)
75–84 years	723 (24.8)	337 (25.7)	386 (24.1)
≥ 85 years	143 (25.9)	50 (26.0)	93 (25.8)
**Age at first birth**^**a**^			
< 20 years	189 (10.4)	47 (15.3)	142 (9.5)
20 – 29 years	2 295 (11.1)	1 107 (14.1)	1 188 (9.3)
30 – 39 years	767 (11.0)	508 (13.1)	259 (8.4)
40 – 49 years	79 (11.2)	61 (12.3)	18 (8.7)
≥ 50 years	9 (17.0)	7 (15.2)	2 (28.6)
**Health status until age 15**^**b**^			
Excellent	1 139 (9.7)	680 (13.0)	459 (7.1)
Very good	1 108 (10.3)	594 (13.1)	514 (8.3)
Good	967 (12.0)	491 (14.6)	476 (10.1)
Fair	294 (13.5)	141 (16.0)	153 (11.8)
Poor	106 (14.0)	41 (14.7)	65 (13.7)
Health varied a great deal	15 (14.2)	5 (13.5)	10 (14.5)
Don’t know	39 (28.5)	22 (27.5)	17 (29.8)
**Education (ISCED 97)**^**c**^			
0—Pre-primary education	252 (16.6)	123 (19.5)	129 (14.5)
1—Primary education	1 111 (16.7)	521 (19.6)	590 (14.8)
2—Lower secondary education	790 (11.4)	414 (14.5)	376 (9.2)
3—Upper secondary education	1 249 (8.7)	736 (11.4)	513 (6.4)
4—Post-secondary non-tertiary education	175 (9.1)	93 (11.2)	82 (7.5)
5—First stage of tertiary education	760 (7.7)	444 (10.3)	316 (5.7)
6—Second stage of tertiary education	26 (6.9)	19 (9.1)	7 (4.2)
No degree/ other	24 (12.6)	13 (16.9)	11 (9.7)
**Marital status**^**d**^			
Married and living together with spouse	3 023 (9.9)	1 856 (13.3)	1 167 (7.1)
Registered partnership	52 (6.9)	31 (8.8)	21 (5.2)
Married, living separated from spouse	58 (10.0)	34 (12.8)	24 (7.7)
Never married	296 (10.8)	161 (12.0)	135 (9.6)
Divorced	313 (8.1)	149 (10.4)	164 (6.8)
Widowed	657 (18.2)	139 (21.3)	518 (17.5)
**Region (as defined in methods)**			
Northern Europe	589 (10.7)	317 (13.1)	272 (8.8)
Southern Europe	1 194 (11.5)	655 (14.7)	539 (9.1)
Western Europe	1 571 (9.3)	862 (12.0)	709 (7.3)
Eastern Europe	1 054 (11.4)	541 (13.5)	513 (9.8)

^a^Missings: 11 943 (Men: 5 500; Women: 6 443)

^b^Missings: 8 370 (Men: 3 700; Women: 4 670)

^c^Missings: 172 (Men: 64; Women: 108)

^d^Missings: 79 (Men: 30; Women: 49)

**Fig. 1:**
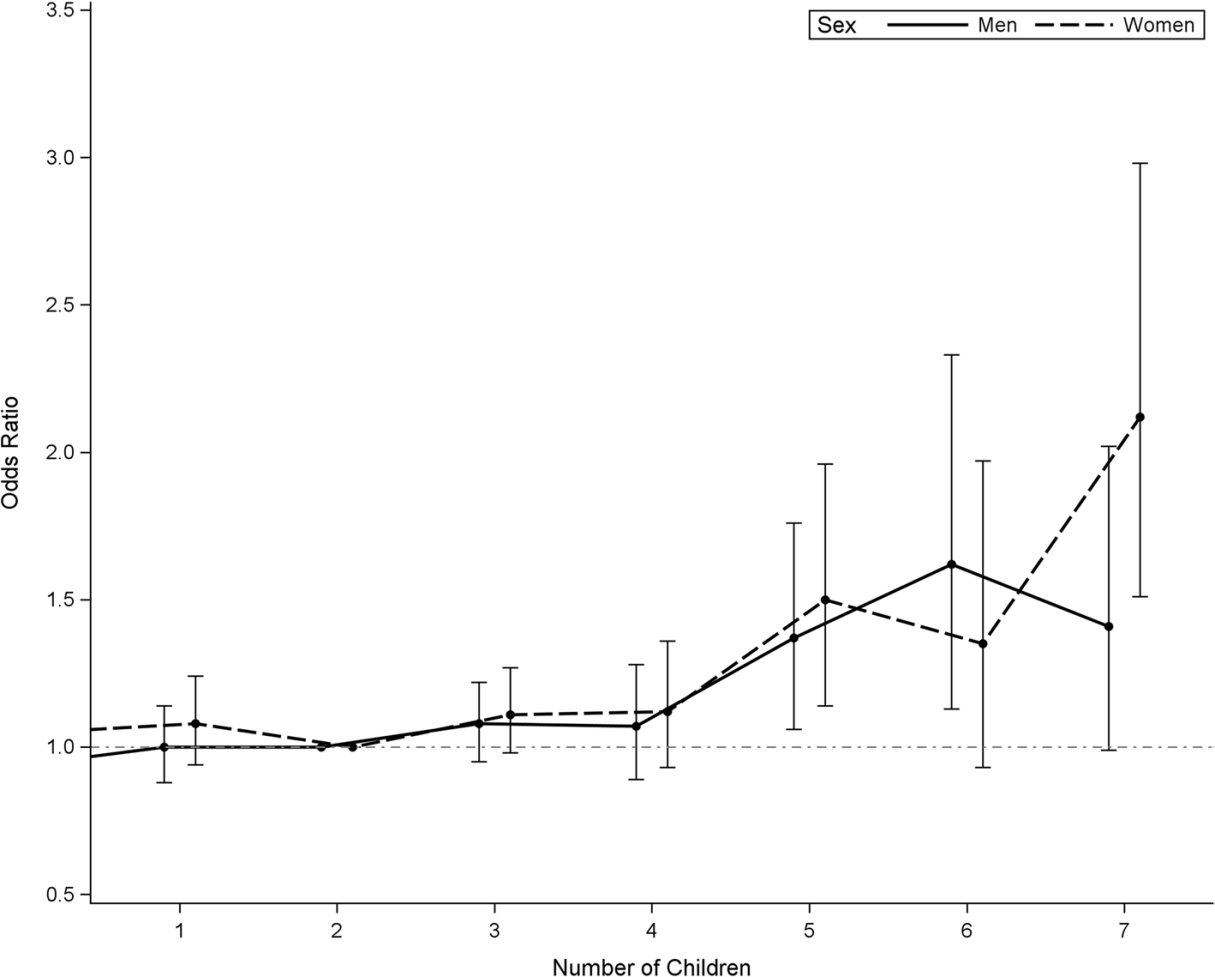
Estimated ORs for the association between number of children and incident HDS. ORs were estimated for 41 699 men and women of the Survey of Health, Ageing and Retirement in Europe (SHARE) from 2004 to 2017. Shown are sex-specific odds ratios of Model 2 (main model) adjusted for baseline age, education, region, and marital status

Men and women with three or more children showed increasing ORs for incident HDS with every additional child, compared to subjects with two children (Fig. 1; Table 3). While for women, the group of subjects with seven or more children showed the highest OR (main model: OR = 2.12 [95% CI: 1.51 to 2.98]), for men, the group of subjects with six children showed the highest OR (OR = 1.62 [1.13 to 2.33]). For five or more children, the exposure categories had only a small number of subjects. For this reason, an additional analysis was conducted for the subjects with five or more children. The ORs for incident HDS for the main model were 1.43 [1.19 to 1.73] for men and 1.60 [1.32 to 1.95] for women.

**Table 3:** Odds ratios for the association between number of children and incident HDS

**Number of children**	**All**			**Men**^**a**^			**Women**^**a**^		
	**N Observations (N Cases)**	**OR**	**95% CL**	**N Observations (N Cases)**	**OR**	**95% CL**	**N Observations (N Cases)**	**OR**	**95% CL**
**Crude Model**	42,075 (4,408)			18,080 (2,375)			23,995 (2,033)		
0	4,175 (459)	1.18	[1.05; 1.31]	1,996 (243)	0.98	[0.84; 1.14]	2,179 (216)	1.40	[1.19; 1.64]
1	7,239 (756)	1.11	[1.01; 1.22]	2,944 (377)	1.04	[0.91; 1.18]	4,295 (379)	1.23	[1.08; 1.40]
2	17,633 (1,676)	Ref		7,603 (943)	Ref		10,030 (733)	Ref	
3	8,141 (859)	1.12	[1.03; 1.23]	3,427 (466)	1.11	[0.99; 1.25]	4,714 (393)	1.15	[1.02; 1.31]
4	2,883 (331)	1.24	[1.09; 1.40]	1,232 (176)	1.18	[0.99; 1.40]	1,651 (155)	1.31	[1.10; 1.58]
5	1,073 (153)	1.58	[1.33; 1.89]	481 (84)	1.49	[1.17; 1.91]	592 (69)	1.67	[1.29; 2.18]
6	471 (80)	1.95	[1.52; 2.49]	194 (42)	1.95	[1.38; 2.77]	277 (38)	2.02	[1.42; 2.86]
≥ 7	460 (94)	2.45	[1.94; 3.09]	203 (44)	1.95	[1.39; 2.75]	257 (50)	3.07	[2.23; 4.21]
**Model 1**	42,075 (4,408)			18,080 (2,375)			23,995 (2,033)		
0	4,175 (459)	1.07	[0.96; 1.20]	1,996 (243)	1.00	[0.86; 1.17]	2,179 (216)	1.13	[0.96; 1.33]
1	7,239 (756)	1.05	[0.95; 1.15]	2,944 (377)	1.00	[0.88; 1.14]	4,295 (379)	1.09	[0.95; 1.24]
2	17,633 (1,676)	Ref		7,603 (943)	Ref		10,030 (733)	Ref	
3	8,141 (859)	1.09	[1.00; 1.20]	3,427 (466)	1.08	[0.96; 1.22]	4,714 (393)	1.11	[0.98; 1.27]
4	2,883 (331)	1.13	[0.99; 1.28]	1,232 (176)	1.10	[0.92; 1.31]	1,651 (155)	1.15	[0.95; 1.39]
5	1,073 (153)	1.44	[1.20; 1.73]	481 (84)	1.37	[1.07; 1.76]	592 (69)	1.54	[1.17; 2.02]
6	471 (80)	1.68	[1.30; 2.16]	194 (42)	1.77	[1.24; 2.52]	277 (38)	1.57	[1.09; 2.27]
≥ 7	460 (94)	1.92	[1.51; 2.45]	203 (44)	1.62	[1.14; 2.29]	257 (50)	2.28	[1.63; 3.19]
**Model 2** **(main model)**	41,699 (4,363)			17,934 (2,350)			23,765 (2,013)		
0	4,121 (453)	0.99	[0.86; 1.13]	1,979 (241)	0.93	[0.78; 1.13]	2,142 (212)	1.05	[0.87; 1.27]
1	7,188 (749)	1.04	[0.94; 1.14]	2,923 (373)	1.00	[0.88; 1.14]	4,265 (376)	1.08	[0.94; 1.24]
2	17,493 (1,661)	Ref		7,547 (934)	Ref		9,946 (727)	Ref	
3	8,068 (853)	1.09	[1.00; 1.19]	3,402 (463)	1.08	[0.95; 1.22]	4,666 (390)	1.11	[0.98; 1.27]
4	2,854 (325)	1.09	[0.96; 1.24]	1,218 (172)	1.07	[0.89; 1.28]	1,636 (153)	1.12	[0.93; 1.36]
5	1,059 (153)	1.42	[1.18; 1.71]	475 (84)	1.37	[1.06; 1.76]	584 (69)	1.50	[1.14; 1.96]
6	465 (77)	1.49	[1.15; 1.93]	191 (41)	1.62	[1.13; 2.33]	274 (36)	1.35	[0.93; 1.97]
≥ 7	451 (92)	1.73	[1.35; 2.22]	199 (42)	1.41	[0.99; 2.02]	252 (50)	2.12	[1.51; 2.98]

Crude Model: unadjusted

Model 1: adjusted for baseline age and sex

Model 2 (main model): adjusted for baseline age, sex, education, region, and marital status

*Abbreviation*: *OR* odds ratio, *CL* confidence limit

^a^stratified analyses not adjusted for sex

Estimating the association between number of children and incident HDS without subjects who were taking only blood pressure or cholesterol medication at first report showed similar increasing ORs for incident HDS with every additional child, compared to subjects with two children, and was highest in the group of participants with 7 or more children in men and women (OR = 1.50 [1.13 to 1.98] and OR = 2.24 [1.76 to 2.85], respectively) (Supplementary Table 2). Similar results were obtained in separate analyses for both heart disease and stroke as outcomes. For both heart disease and stroke as separate outcomes, the highest ORs were observed in the group of people with seven or more children (OR = 1.68 [1.29 to 2.19] and OR = 2.01 [1.38 to 2.92], respectively) (Supplementary Table 3).

Further analyses stratified by education level showed the highest ORs for the association between the number of children and incident HDS across all education levels in the group with five or more children compared to those with one or two children (Table 4). The highest ORs for the group with five or more children were observed in both women and men in the group with primary education (OR = 1.66 [1.29 to 2.15] and OR = 1.60 [1.19 to 2.14], respectively). In analyses stratified by region, the highest ORs were also observed in the group of individuals with five or more children compared to those with one or two children, except for men from Eastern Europe (OR = 1.02 [0.64 to 1.65]). Among women with five or more children, the lowest ORs were observed in Northern Europe (OR = 1.27 [0.73 to 2.19]). For both men and women with five or more children, the highest ORs were observed in Southern Europe (OR = 2.07 [1.52 to 2.82] and OR = 1.75 [1.25 to 2.44], respectively).

**Table 4:** Odds ratios for incident HDS by number of children stratified by education and region

	Number of children	**Men**	OR [95% CL]	**Women**	OR [95% CL]
		N Observations (N Cases)		N Observations (N Cases)	
**Education (ISCED 97)**					
Primary (ISCED 0–1)		3 291 (644)		4 865 (719)	
	0	404 (65)	0.75 [0.50; 1.12]	405 (67)	0.90 [0.64; 1.26]
	1–2	1 641 (304)	Ref	2 555 (350)	Ref
	3–4	962 (197)	1.11 [0.91; 1.36]	1 446 (203)	1.01 [0.84; 1.23]
	≥ 5	284 (78)	1.60 [1.19; 2.14]	459 (99)	1.66 [1.29; 2.15]
Secondary (ISCED 2–4)		10 127 (1,243)		13 168 (971)	
	0	1 092 (128)	1.00 [0.79; 1.28]	1 120 (103)	1.04 [0.81; 1.35]
	1–2	6 187 (741)	Ref	8 273 (578)	Ref
	3–4	2 431 (308)	1.06 [0.92; 1.22]	3 311 (250)	1.12 [0.95; 1.31]
	≥ 5	417 (66)	1.35 [1.02; 1.79]	464 (40)	1.32 [0.93; 1.86]
Tertiary (ISCED 5–6)		4 516 (463)		5 732 (323)	
	0	483 (48)	0.95 [0.65; 1.38]	617 (42)	1.10 [0.73; 1.66]
	1–2	2 642 (262)	Ref	3 383 (175)	Ref
	3–4	1 227 (130)	1.08 [0.86; 1.35]	1 545 (90)	1.13 [0.86; 1.49]
	≥ 5	164 (23)	1.35 [0.85; 2.16]	187 (16)	1.50 [0.86; 2.60]
**Region**					
North		2 398 (313)		3 077 (268)	
	0	203 (21)	0.56 [0.32; 1.01]	206 (21)	0.92 [0.55; 1.56]
	1–2	1 306 (170)	Ref	1 720 (144)	Ref
	3–4	760 (101)	1.01 [0.77; 1.32]	991 (86)	1.03 [0.77; 1.37]
	≥ 5	129 (21)	1.31 [0.79; 2.16]	160 (17)	1.27 [0.73; 2.19]
South		4 409 (647)		5 872 (532)	
	0	498 (65)	0.84 [0.56; 1.25]	597 (59)	0.89 [0.60; 1.30]
	1–2	2 629 (352)	Ref	3 532 (283)	Ref
	3–4	1 036 (159)	1.02 [0.83; 1.26]	1 428 (134)	1.05 [0.84; 1.32]
	≥ 5	246 (71)	2.07 [1.52; 2.82]	315 (56)	1.75 [1.25; 2.44]
West		7 136 (851)		9 594 (700)	
	0	955 (113)	1.04 [0.80; 1.34]	1 040 (100)	1.20 [0.92; 1.58]
	1–2	3 926 (440)	Ref	5 501 (353)	Ref
	3–4	1 923 (245)	1.13 [0.95; 1.33]	2 636 (195)	1.12 [0.93; 1.35]
	≥ 5	332 (53)	1.24 [0.90; 1.70]	417 (52)	1.56 [1.12; 2.16]
East		3 991 (539)		5 222 (513)	
	0	323 (42)	1.03 [0.68; 1.56]	299 (32)	0.90 [0.59; 1.38]
	1–2	2 609 (345)	Ref	3 458 (323)	Ref
	3–4	901 (130)	1.10 [0.88; 1.38]	1 247 (128)	1.12 [0.89; 1.40]
	≥ 5	158 (22)	1.02 [0.64; 1.65]	218 (30)	1.43 [0.94; 2.18]

Education: adjusted for baseline age, region, marital status

Region: adjusted for baseline age, education, marital status

*Abbreviation*: *OR* odds ratio, *CL* confidence limit

## Discussion

In the present study, we found an association between the number of children and incident HDS, with the highest odds in parents with five children or more compared to those with two children. This association was + more pronounced in men and women with primary education and subjects living in Southern Europe, and less strong in women living in Northern Europe and men living in Eastern Europe.

Our findings are in line with previous studies showing that multiparous individuals are at greater risk for cardiovascular diseases [5–13]. However, we could not find a J-shaped association as described in some previous studies [5–8], which also seems plausible. A higher odds of heart disease in those without children may be due to a higher potential for illness in later life. Children could thus be supportive of better health in old age. Our study also confirms the few studies in men, that observed an increased risk of cardiovascular disease with increasing numbers of children in fathers [5, 8–10]. A recent American study by Hipp et al. [9], following 24 923 individuals aged 50 years and older over a period of 22 years, found an association between incident heart disease and number of children, showing the highest hazard ratios (HR) in the group of five children or more (men: HR = 1.07 [95% CI: 0.95 to 1.20]; women: HR = 1.13 [1.03 to 1.25]), which is similar to our results.

In our study, we observed that the ORs of incident HDS differed between educational groups. While women with five or more children and the lowest educational level had the highest OR (OR = 1.66 [95% CI: 1.29 to 2.15]), we found no substantial differences between secondary and tertiary educational levels. The results were even statistically significant for both men and women in the lowest education group with five or more children compared to those with only one or two children. This observation may be explained by lower resources in the group with the lowest educational level. A high number of children may lead to interruptions in educational and occupational careers, resulting in lower financial resources and thus increased physical and psychological stress and cardiovascular risk [3, 4, 25–27].

Interestingly, in our study, we found regional variations in the ORs of HDS. Among men and women with five or more children, those living in Southern Europe had a statistically significant higher OR of HDS compared to those with one or two children, while women living in Northern Europe and men living in Eastern Europe had less strong associations. A possibly better work-life balance, social, and financial support, as well as higher education for women in Scandinavian countries could account for the less strong ORs of cardiovascular disease observed in our study compared with the other regions considered. In addition, the ORs of incident HDS differs less between men and women in Northern Europe compared to other regions, which may indicate greater equality between the sexes. In contrast, social norms in child rearing, lower financial support, and greater gender inequality might explain the higher ORs in Southern Europe.

However, having a large family can also have a positive impact on parents' health. A larger family provides more social interaction as well as instrumental and emotional support. A study by Steptoe et al. [28] examined blood pressure during workdays in participants with different family structures. They found that there was no difference during workdays among participants, but parents showed a greater day-evening decrease in systolic blood pressure. This decrease was moderated primarily by social support. Parents experiencing high levels of social support showed the greatest difference. This observation did not differ between men and women and indicated a cardioprotective effect of a supportive family life. In addition, children provide an incentive for a healthier lifestyle for parents through an increased sense of responsibility and social pressure to behave adequately. However, the positive aspects of better health behaviors relate primarily to a family size within the norm. An increase in the number of children may have a negative impact on parents' health behaviors, as they may pay less attention to their own needs [29]. A study by Van den Broek et al. [2] highlighted a positive relationship between the number of children and maternal BMI. Mothers of three or more children had an 18.3 percentage point higher predicted probability of being overweight than women with two children. For obesity, the predicted probability was 8.6 percentage points higher compared to mothers with two children.

A strength of our study is the harmonized design and large study size, which allow us to examine the research question with a large number of people from 20 different countries. However, since this is an observational study, it is not possible to conclude a causal relationship. Residual confounding can also not be excluded. Due to the formulation of the question, we were not able to distinguish between own, adopted, or stepchildren. For this reason, the reported number of children probably overestimates family size. Furthermore, the outcome was only self-reported. However, studies on the reliability of self-reported myocardial infarction and stroke show high sensitivity and specificity, whereas the agreement between self-reported and physician-diagnosed heart failure is lower [30]. Due to the fact that self-report of heart diseases requires participants to have survived the outcome, the study population tends to reflect a healthier group than the general population. This could lead to an underestimation of the effect. However, the use of end-of-life interviews counteracts this effect.

## Conclusion

Our study adds to the picture of an association between the number of children and incident cardiovascular events. In our European cohort, we observed an association in both sexes that may be modulated by factors related to educational level and region. Elucidation of the biological association and further prospective studies examining more social covariates, as well as regional comparisons, are needed to understand the mechanisms for the increased cardiovascular risk associated with an increased number of children.

### Supplementary Information

**Additional file 1:** **Supplementary Figure 1.** Selection of the study population. **Supplementary Table 1.** Proportion of individuals with variation in the number of children reported. **Supplementary Table 2.** Odds ratios for the association between number of children and incident HDS without exclusion of participants who were taking only blood pressure or cholesterol medication at first report. **Supplementary Table 3.** Odds ratios for the association between number of children and incident heart disease as well as incident stroke separately.

## Data Availability

Data is publicly available upon application (see https://www.share-project.org).

## Abbreviations

SHARE: Survey of Health, Ageing and Retirement in Europe
OR: Odds ratio
HDS: Heart disease and/or stroke
95% CI: 95% Confidence interval
ISCED 97: 1997 International Standard Classification of Education
HR: Hazard ratios
