# Supplementary material for: Association between number of children and incident heart disease and stroke in parents – results from the Survey of Health, Ageing and Retirement in Europe (SHARE)
Source: BMC Public Health. 2023 Nov 24;23:2324. doi: 10.1186/s12889-023-17254-7 (PMC10668373; doi:10.1186/s12889-023-17254-7)
Supplement: Supplementary file 1 — Additional file 1: Supplementary Figure 1. Selection of the study population. Supplementary Table 1. Proportion of individuals with variation in the number of children reported. Supplementary Table 2. Odds ratios for the association between number of children and incident HDS without exclusion of participants who were taking only blood pressure or cholesterol medication at first report. Supplementary Table 3. Odds ratios for the association between number of children and incident heart disease as well as incident stroke separately. [file 12889_2023_17254_MOESM1_ESM.docx]

**
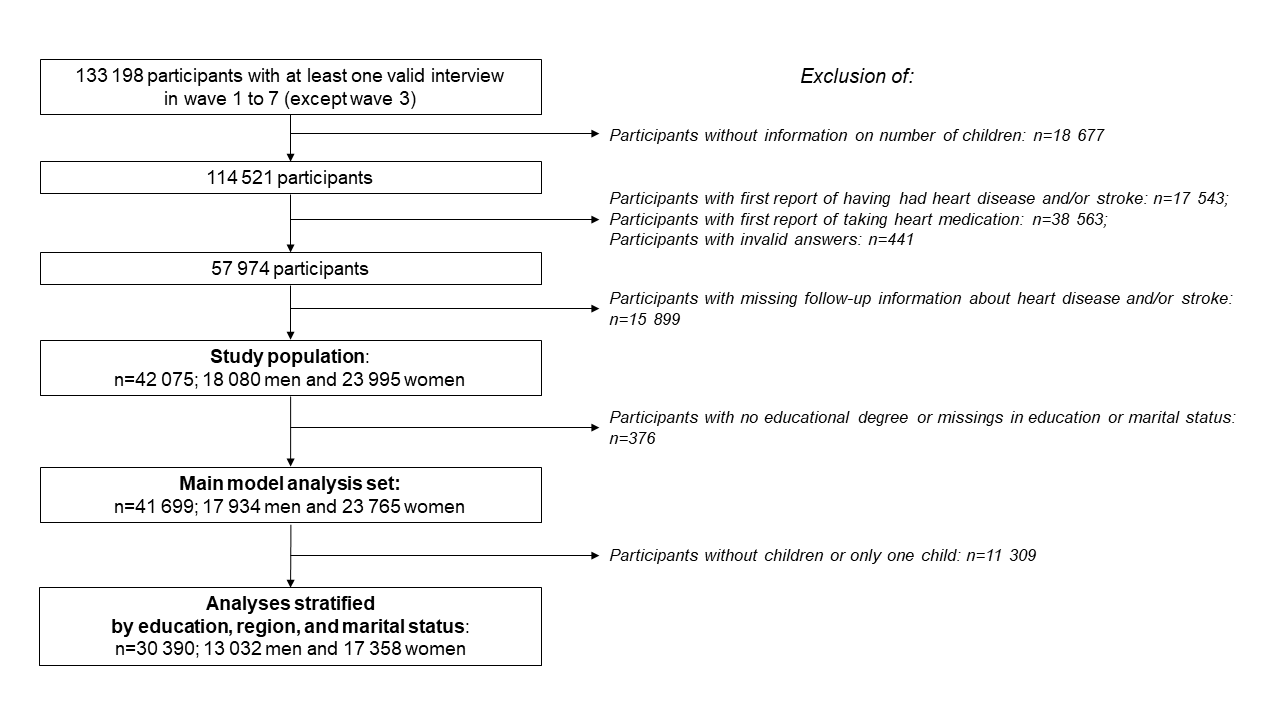
**

**Supplementary Figure 1: Selection of the study population.**

**Supplementary Table 1: Proportion of individuals with variation in the number of children reported.**

|  | N (=42,075) | % |
| --- | --- | --- |
| Variation in reported number of children | 4,911 | 11.7 |
| No variation in reported number of children | 37,164 | 88.3 |

**Supplementary Table 2: Odds ratios for the association between number of children and incident HDS without exclusion of participants who were taking only** **blood pressure or cholesterol medication at first report.**

|  | All | | | Men* | | | Women* | | |
| --- | --- | --- | --- | --- | --- | --- | --- | --- | --- |
| Number of children | **N Observations**  **(N Cases)** | **OR** | **95% CL** | **N Observations**  **(N Cases)** | **OR** | **95% CL** | **N Observations**  **(N Cases)** | **OR** | **95% CL** |
| Model 2  (main model) | 66,196 (8,682) |  |  | 28,005 (4,304) |  |  | 38,191 (4,378) |  |  |
| 0 Children | 6,294 (840) | 1.03 | [0.93; 1.13] | 2,953 (417) | 0.96 | [0.84; 1.11] | 3,341 (423) | 1.10 | [0.96; 1.26] |
| 1 Child | 11,649 (1,535) | 1.05 | [0.98; 1.12] | 4,694 (702) | 1.00 | [0.91; 1.10] | 6,955 (833) | 1.10 | [1.00; 1.21] |
| 2 Children | 27,858 (3,302) | Ref. |  | 11,894 (1,716) | Ref. |  | 15,964 (1,586) | Ref. |  |
| 3 Children | 12,687 (1,720) | 1.14 | [1.07; 1.21] | 5,287 (871) | 1.13 | [1.03; 1.24] | 7,400 (849) | 1.15 | [1.05; 1.26] |
| 4 Children | 4,582 (664) | 1.15 | [1.05; 1.26] | 1,901 (322) | 1.12 | [0.98; 1.28] | 2,681 (342) | 1.19 | [1.04; 1.35] |
| 5 Children | 1,691 (304) | 1.43 | [1.25; 1.64] | 698 (140) | 1.32 | [1.08; 1.60] | 993 (164) | 1.55 | [1.29; 1.86] |
| 6 Children | 731 (138) | 1.40 | [1.15; 1.70] | 289 (62) | 1.39 | [1.03; 1.86] | 442 (76) | 1.40 | [1.08; 1.83] |
| ≥ 7 Children | 704 (179) | 1.86 | [1.55; 2.23] | 289 (74) | 1.50 | [1.13; 1.98] | 415 (105) | 2.24 | [1.76; 2.85] |

*stratified analyses not adjusted for sex
Model 2 (main model): adjusted for baseline age, sex, education, region, and marital status

**Supplementary Table 3: Odds ratios for the association between number of children and incident heart disease as well as incident stroke separately.**

|  | Heart Disease | | | Stroke | | |
| --- | --- | --- | --- | --- | --- | --- |
| Number of children | **N Observations**  **(N Cases)** | **OR** | **95% CL** | **N Observations**  **(N Cases)** | **OR** | **95% CL** |
| Model 2 (main model) | 41,699 (3,444) |  |  | 41,699 (1,306) |  |  |
| 0 Children | 4,121 (346) | 0.94 | [0.81; 1.09] | 4,121 (151) | 1.15 | [0.92; 1.43] |
| 1 Child | 7,188 (585) | 1.02 | [0.92; 1.13] | 7,188 (248) | 1.18 | [1.01; 1.39] |
| 2 Children | 17,493 (1,326) | Ref. |  | 17,493 (473) | Ref. |  |
| 3 Children | 8,068 (668) | 1.07 | [0.97; 1.18] | 8,068 (247) | 1.08 | [0.92; 1.27] |
| 4 Children | 2,854 (252) | 1.06 | [0.92; 1.23] | 2,854 (92) | 1.03 | [0.82; 1.30] |
| 5 Children | 1,059 (133) | 1.56 | [1.29; 1.90] | 1,059 (38) | 1.14 | [0.81; 1.61] |
| 6 Children | 465 (61) | 1.46 | [1.10; 1.94] | 465 (23) | 1.41 | [0.91; 2.18] |
| ≥ 7 Children | 451 (73) | 1.68 | [1.29; 2.19] | 451 (34) | 2.01 | [1.38; 2.92] |

Model 2 (main model): adjusted for baseline age, sex, education, region, and marital status
